# Image-Enhanced Capsule Endoscopy Improves the Identification of Small Intestinal Lesions

**DOI:** 10.3390/diagnostics11112122

**Published:** 2021-11-15

**Authors:** Noriyuki Ogata, Kazuo Ohtsuka, Masataka Ogawa, Yasuharu Maeda, Fumio Ishida, Shin-ei Kudo

**Affiliations:** 1 Digestive Disease Center, Showa University Northern Yokohama Hospital, Yokohama 224-8503, Japan; kohtsuka.gast@tmd.ac.jp (K.O.); skyscraper@med.showa-u.ac.jp (M.O.); yasuharumaeda610@hotmail.com (Y.M.); fumio182@med.showa-u.ac.jp (F.I.); kudos@med.showa-u.ac.jp (S.-e.K.); 2 Department of Endoscopy, Tokyo Medical and Dental University, Medical Hospital, Tokyo 113-0034, Japan

**Keywords:** capsule endoscopy, image-enhanced endoscopy, flexible spectral color enhancement, contrast capsule, small intestinal lesions

## Abstract

Image-enhanced endoscopy is useful for diagnosing and identifying lesions in the gastrointestinal tract. Recently, image-enhanced endoscopy has become a breakthrough technology that has attracted significant attention. This image enhancing technology is available for capsule endoscopy, which is an effective tool for small intestinal lesions and has been applied in flexible spectral color enhancement technology and in contrast capsule like narrow-band imaging. In this field, most researchers focus on improving the visibility and detection of small intestinal lesions. This review summarizes previous studies on image-enhanced capsule endoscopy and aims to evaluate the efficacy of this technology.

## 1. Introduction

Capsule endoscopy (CE) was introduced in 2000 [1]. CE allows the visualization of the mucosa throughout the small intestine. Several studies have shown that CE is an effective means of detecting lesions in the small bowel, particularly sites of obscure gastrointestinal bleeding [2,3,4,5,6]. CE is also one of the standard modalities for monitoring Crohn’s disease [7,8,9,10,11,12,13]. Since 2004, image-enhanced endoscopy (IEE) has been reported to improve the detection and diagnosis of gastrointestinal lesions [14,15,16]. IEE techniques are useful for enhancing images of vasculature patterns, and for improving the visibility of surface patterns and color differences. Several IEE modalities are used in daily clinical practice, including narrow-band imaging (NBI, Olympus Corp., Tokyo, Japan), flexible spectral color enhancement (FICE, Fujifilm Corp., Tokyo, Japan), blue laser imaging (BLI, Fujifilm Corp., Tokyo, Japan), and linked color imaging (LCI, Fujifilm Corp., Tokyo, Japan) [17,18,19,20,21]. However, the diagnostic efficacy of CE is reduced when the identification of the mucosa is impaired due to the interference of bubbles, food residues, bile, and blood. Although the visibility might be improved by bowel preparation, there are limits to the effectiveness of bowel preparation for CE [22]. Therefore, IEE would be important for improving the identification of small bowel lesions in the presence of bile and blood. Furthermore, there is a problem with the diagnostic yield of CE because small lesions are difficult to detect and diagnose. Zheng et al. reported that the overall positive diagnostic yield of CE for small bowel disease remains at about 60% [23]. Thus, there is a need for IEE to enable the visualization of such lesions. A previous study reported that performing CE using a FICE digital processing system within a capsule workstation (Given Imaging, Yoqneam, Israel) is feasible [24]. Other reports have suggested that FICE may improve the visibility of small intestinal lesions [25,26]. Furthermore, a similar function for CE, developed by Olympus Inc., has shown promising results. The contrast capsule (CC) is an image-enhanced capsule endoscope (IECE) with blue-enhanced white light-emitting diodes (WL-LEDs) that allow high-contrast images to be obtained by selecting predominantly blue and green wavelengths of light [25].

This review aims to consolidate existing clinical data on the utility of IEE for improving the detection rate and characterization of small bowel pathological findings using CE, as compared to that of white light images (WLIs).

## 2. FICE

The FICE system was developed and introduced in 2005 as an image-processing tool for conventional video endoscopy. FICE is a digital processing method that uses WLIs and processes the images by emphasizing specific ranges of wavelengths. Three wavelengths (red, green, and blue) are selected and assigned to construct a complex enhanced image [26]. FICE has been hypothesized to enhance surface patterns and can improve the visualization and detection of lesions [27]. FICE is used in gastroscopy and colonoscopy to increase the detection rate of neoplastic lesions.

**Figure 1:**
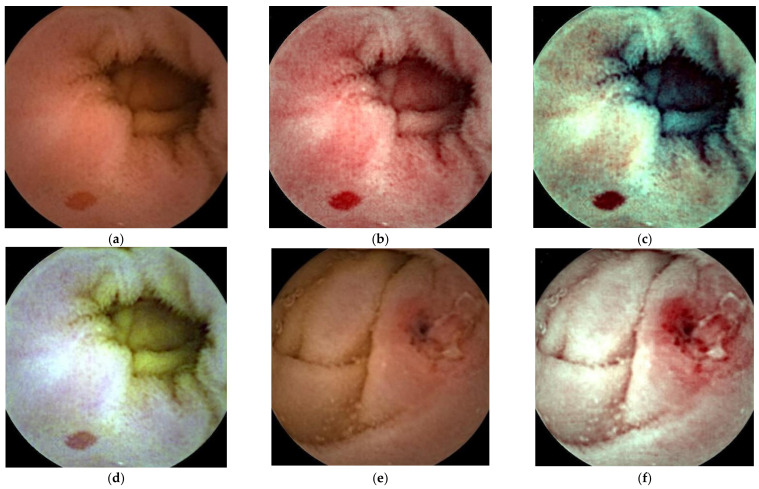

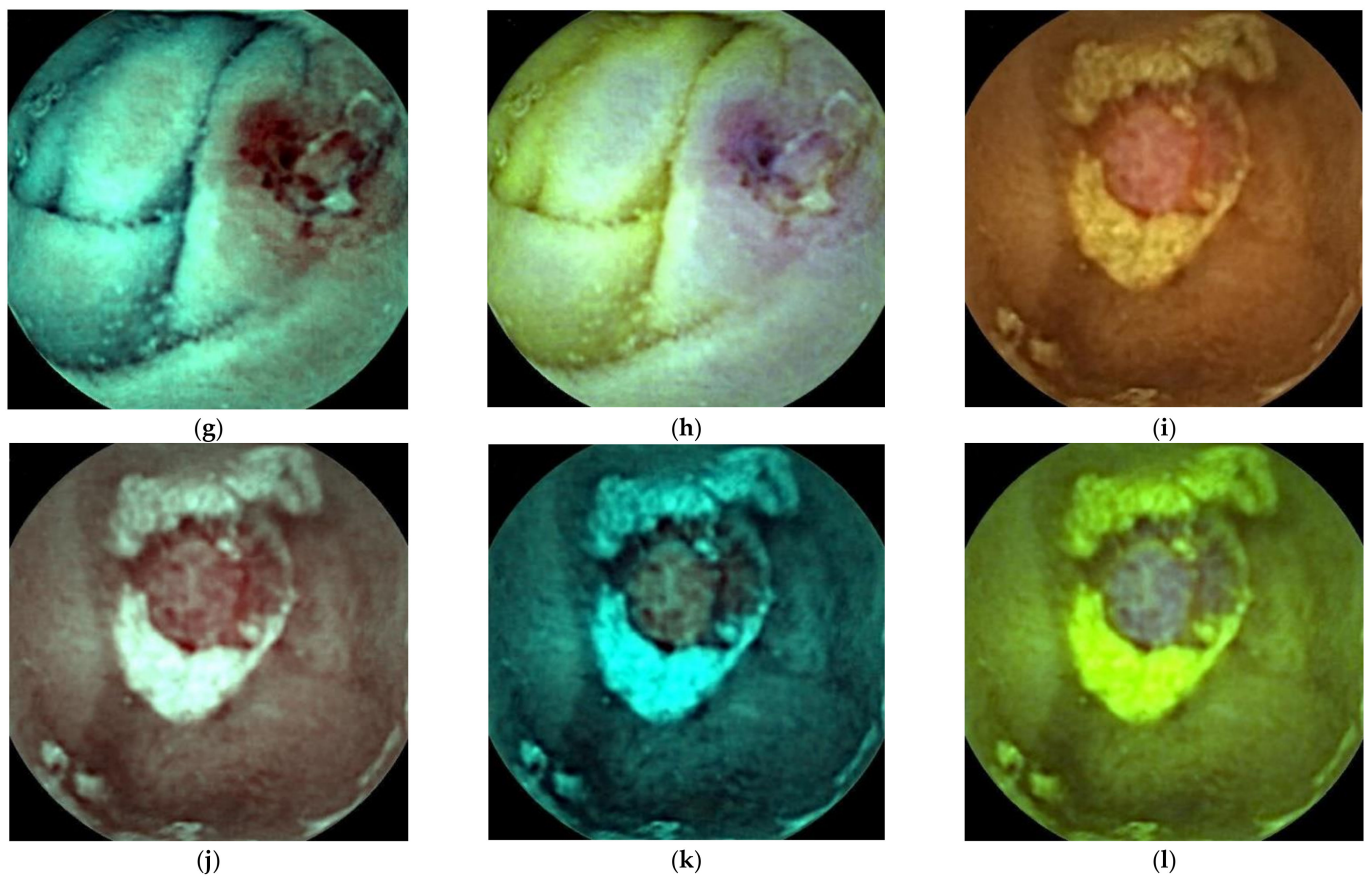
Capsule endoscopy images of small intestinal lesions; angioectasia (**a**–**d**), ulcer/erosion (**e**–**h**), tumor (**i**–**l**). WLI (**a**,**e**,**i**), FICE 1(**b**,**f**,**j**), FICE 2 (**c**,**g**,**k**), FICE 3 (**d**,**h**,**l**).

FICE is a digital image technology that uses the RAPID software (Given Imaging Ltd., Yoqneam, Israel), which enables the processing of regular images captured by normal video capsule devices (Figure 1). The FICE wavelength settings are as follows: FICE 1 (red, 595 nm; green, 540 nm; blue, 535 nm); FICE 2 (red, 420 nm; green, 520 nm; blue 530 nm); and FICE 3 (red, 595 nm; green, 570 nm; blue, 415 nm). FICE 1 aims to reduce bile interference, FICE 2 aims to emphasize blood, and FICE 3 aims to emphasize the differences between bile and blood.

## 3. Contrast Capsule

**Figure 2:**
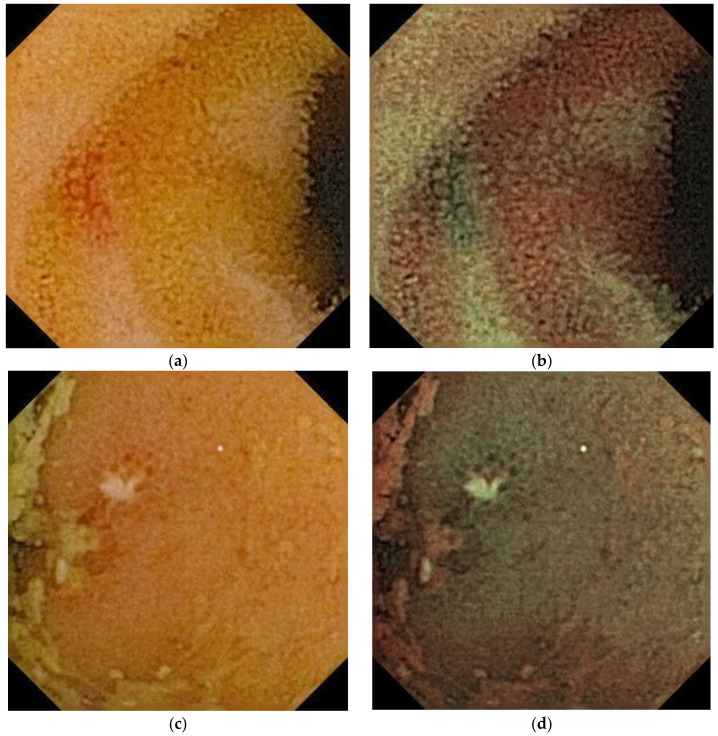
Capsule endoscopy images of small intestinal lesions: angioectasia (**a**,**b**) and ulcer/erosion (**c**,**d**). WLI (**a**,**c**), CC (**b**,**d**).

A CC is an image-enhanced CE. This is a normal capsule (EC type1, Olympus Corp.) equipped with a special WL-LED that was selected to increase the level of brightness in the blue wavelength range, which is appropriate for the visualization of hemoglobin. WLIs are generated using data from the three primary colors (red, green, and blue), conventionally obtained from a charge-coupled device. Contrast images (CIs) are generated from intentionally selecting the green and blue data (Figure 2). This technology allows for stronger color contrast between hemoglobin and normal mucosa, potentially contributing to the detection of bleeding points. CC is classified as an optical-digital method, similar to NBI in the endoscopic imaging classification [28]. In 2011, Aihara et al. reported that CC visualized the color contrast between normal mucosa and small intestinal lesions, such as angioectasia, erosion/ulcer, and polyps [29].

## 4. Detection of Lesions

Previous studies have shown that FICE improves the detection rate of small intestinal lesions, with FICE 1 and FICE 2 suited to the detection of erosions, ulcers, and angioectasia (Table 1). Imagawa et al. [30] reported that FICE 1 and FICE 2 had higher levels of angioectasia detection. In their study, two experienced endoscopists analyzed 50 videos. One endoscopist read the images obtained by conventional CE, and the other, blinded to the results of the conventional readings, read the images obtained from FICE at settings 1, 2, and 3. Seventeen angioectasias were identified by conventional WLI. Forty-eight were detected by FICE 1, 45 by FICE 2, and 24 by FICE 3, with significant differences at FICE 1 and 2 (*p* = 0.0003 and *p* < 0.0001, respectively). There was no difference between conventional WLI and FICE for the detection of erosions, ulcerations, and tumors. However, Gupta et al. [31] reported that FICE did not significantly improve the detection of small intestinal lesions compared with WLIs. In their study, two gastrointestinal fellows retrospectively analyzed 60 CE examinations with and without FICE. The senior consultant evaluated findings as P0, P1, and P2 lesions (non-pathological, intermediate bleeding potential, and high bleeding potential, respectively), considered as reference findings. A total of 153 lesions were diagnosed by the two fellows with FICE as compared to 118 with WLIs (*p* = 0.15). With FICE, the sensitivity and specificity of the detection rate of P2 lesions were 94% (0.87–1.02) and 95% (0.87–1.03), respectively, and with WLI, they were 97% (0.92–1.02) and 96% (0.86–1.04) respectively. There was no difference between conventional WLI and FICE for P2 lesions. Significantly more P0 lesions were diagnosed by the two fellows using FICE as compared to WLIs (39 vs. 8, *p* < 0.01). In these reports, FICE was not useful in detecting tumors. Kobayashi et al. [32] reported that FICE 1 was better at detecting angioectasias and ulcerative lesions, and worse at detecting tumors or polyps compared to WLIs. In their study, three endoscopists analyzed 24 CE examinations with conventional WLI and three settings of FICE and compared their sensitivity and specificity for the detection of small intestinal lesions. Significantly more angioectasias and ulcerative lesions were detected with FICE 1 as compared to WLIs (angioectasia, 25.7 vs. 21.0, *p* = 0.005; ulcerative lesions, 19.3 vs. 14.0, *p* = 0.06). However, significantly more tumors were missed with FICE 1 as compared to WLIs (4.3 vs. 10.0, *p* = 0.003). Sato et al. [33] similarly showed that compared with WLIs, FICE 1 was better at detecting angioectasias and ulcerative lesions, FICE 2 was better at detecting angioectasias, and each FICE setting was ineffective at detecting tumors or polyps. In their study, to compare the detection rate of small bowel lesions using images obtained from the three settings of FICE or blue mode with images obtained from WLI, a total of 50 patients who underwent CE were enrolled. CE was performed for the following reasons: obscure gastrointestinal bleeding (*n* = 34), examination of the extent of tumor spread (*n* = 8), investigation into the source of chronic abdominal pain or diarrhea (*n* = 4), and miscellaneous (*n* = 4). Three expert endoscopists who had similar levels of experience in CE analyzed the videos. A first endoscopist analyzed videos 1 to 20 with WLI, videos 21 to 40 with the three types of FICE, and videos 41 to 50 with blue mode. A second endoscopist analyzed the same sequences of videos using FICE, blue mode, and WLI, sequentially, and a third endoscopist analyzed the videos using blue mode, WLI, and FICE, sequentially. The FICE reader reviewed the three settings of FICE on a different day in a blinded fashion. The relevant findings obtained from CE were documented and classified by each endoscopist as vascular lesion, erosion/ulceration, tumor, or no abnormality. The numbers of detected lesions and period of evaluation were compared between WLI, the three settings of FICE, and blue mode. The final diagnoses determined by several modalities including CE, balloon-assisted enteroscopy, surgery, and periodical observation, were used as the gold standard for this analysis. In this study, 17 angioectasias were identified with WLI; 24 were detected with FICE 1, 33 with FICE 2, and 18 with FICE 3; and 20 were detected with blue mode. There were significant differences between WLI and FICE settings 1 and 2 (*p* = 0.02 and *p* = 0.003, respectively). A total of 28 erosions/ulcers were detected with WLI; 33 with FICE 1, 41 with FICE 2, 24 with FICE 3; and 28 with blue mode. Only FICE 2 showed a significantly higher level of detection ability as compared to WLI (*p* = 0.007). For tumors, a total of 13 lesions were detected with WLI; 13 were detected with FICE 1, 14 with FICE 2, and 10 with FICE 3; and 14 were detected with blue mode. The number of tumors detected did not differ significantly.

Regarding CC, Ogata et al. [25] reported that CIs improved the detection rate of erosions, ulcers, and angioectasia compared to WLIs. In this study, 24 CE videos were evaluated by two trainee endoscopists; one evaluated video CIs and the other evaluated video WLIs in a blinded fashion. The numbers of lesions detected by CIs and WLIs were compared. Of a total of 107 erosions or ulcers, 98 were identified by CIs and 72 were detected by WLIs (*p* < 0.001). Of the 31 angioectasias, 28 were identified by CIs and 20 were detected by WLIs (*p* = 0.015). As with FICE, Hatogai et al. showed that the number of detected polyps and the diagnostic accuracy did not differ significantly between CIs and WLIs [39].

## 5. Characterization of Lesions

In most reports, FICE was useful for improving the ability to diagnose small intestinal lesions (Table 2). Imagawa et al. [40] reported that FICE improved the visibility of small bowel tumors, angioectasias, erosions, and ulcers compared to WLIs, especially with the use of FICE 1 and FICE 2. They also showed that FICE was particularly useful for improving the visibility of hemangioma. In their study, five physicians compared FICE images with corresponding WLIs of 145 lesions obtained from 122 patients. The lesions were categorized as angioectasias (*n* = 23), erosions/ulcerations (*n* = 45), or tumors (*n* = 75). Physicians scored FICE images for the visibility of the lesions according to the following scale: “+2” (improved visibility), “+1” (somewhat improved visibility), “0” (visibility equivalent to that of conventional video CE visibility), “−1” (somewhat decreased visibility), and “−2” (decreased visibility). With FICE 1, improvement was observed for 87% (20/23) of the angioectasia images, 53.3% (26/47) of the erosion/ulceration images, and 25.3% (19/75) of the tumor images; with FICE 2, improvement was observed for 87% (20/23), 25.5% (12/47), and 20.0% (15/75) of images, respectively. With FICE 3, only equivalence was observed. Intra-observer agreement was 0.678 for FICE 1, 0.542 for FICE 2, and 0.597 for FICE 3. However, Krystallis et al. [41] showed that the benefit of FICE for small intestine lesions is limited. In their study, two physicians compared FICE images with corresponding WLIs of 167 lesions obtained from 200 patients. Physicians scored FICE images for the visibility of the lesions according to the following scale: improved visibility, similar visibility, worse visibility. With FICE 1, improvement was observed for 77.7% of the angioectasia images, 36.6% of the erosion/ulceration images, and 14.7% of the tumor images. With FICE 2, improvement was observed for 27.7% of the angioectasia images, 3% of the erosion/ulceration images, and 5.8% of the tumor images. With FICE 3, improvement was observed for 5.5% of the angioectasia images, 3% of the erosion/ulceration images, and 14.7% of the tumor images. This suggests that FICE 1 is effective for angioectasia and only partially effective for ulcer/erosion, and FICE 2 and FICE 3 are ineffective for improving small intestinal lesion images. Inter-observer agreement was 0.646 for FICE 1, 0.617 for FICE 2, and 0.669 for FICE 3. Cotter et al. [42] showed FICE can improve the delineation of angioectasias, ulcers/erosions, and villous edema/atrophy. In their study, two physicians compared FICE images with corresponding WLIs of 100 lesions obtained from 49 patients. Physicians scored the delineation of the lesions on FICE images as follows: improved visibility, similar visibility, worse visibility. Improving the delineation of lesions was achieved in 77% of cases with FICE 1, 74% with FICE 2, and 41% with FICE 3, with a percentage of agreement between investigators of 89% (κ = 0.833), 85% (κ = 0.764), and 66% (κ = 0.486), respectively. With FICE 1, improvement of the delineation was achieved for 97.4% of the angioectasias, 63.3% of the ulcers/erosions, and 66.7% of the villous edema/atrophy. With FICE 2, improvement of the delineation was achieved for 97.4% of the angioectasias, 57.1% of the ulcers/erosions, and 66.7% of the villous edema/atrophy. With FICE 3, improvement of the delineation was achieved for 46.2% of the angioectasias, 24.5% of the ulcers/erosions, and no cases of villous edema/atrophy. Chetcuti Zammit et al. [43] reported that FICE was not useful compared with conventional WLI for the delineation of mucosal changes related to celiac disease. In their study, five expert reviewers evaluated several normal and abnormal CE images obtained from the patients with celiac disease to determine whether the use of FICE can improve the detection of celiac disease-related changes. With conventional WLI, the sensitivity and specificity in the delineation of celiac disease-related changes were 100%. With FICE 1, the sensitivity was 80% and the specificity was 100%. There was no difference between conventional WLI and FICE for the identification of celiac disease-related changes. Inter-rater reliability was low (Fleiss kappa 0.107; *p* = 0.147) between expert reviewers in selecting the best image modification.

Regarding CC, Ogata et al. [25] suggested that CIs obtained using CE improve the visibility of small bowel erosions, ulcers, and angioectasias compared to WLIs. In their study, three physicians retrospectively compared the CIs with the corresponding WLIs of 137 lesion images obtained from 24 patients. For erosions and ulcers, CIs were considered to achieve better visibility in 27.1% of the cases (29/107), equivalent visibility in 63.6 % of the cases (68/107), and inferior visibility in 9.3% of the cases (10/107) (*p* = 0.024). For areas of angioectasia, CIs were considered to achieve better visibility in 48.4% of the cases (15/31), equivalent visibility in 41.9% of the cases (13/31), and inferior visibility in 9.7% of the cases (3/31) (*p* = 0.047). The inter- and intra-observer agreements for the visibility were 0.49 and 0.58, respectively. The lesion images were also investigated by calculating the color difference (ΔE) between the WLIs and CIs using the CIElab color space method. For erosions and ulcers, ΔE for CIs was significantly higher than WLIs (31.9 ± 12.4 compared with 18.7 ± 9.7, respectively; *p* < 0.001). This was also the case for angioectasia (38.4 ± 15.5, compared with 27.1 ± 13.6, respectively; *p* = 0.003). Similarly, Hatogai et al. reported that CIs are an effective tool for enhancing polyp visibility [39].

## 6. Colon Capsule Endoscopy

**Figure 3:**
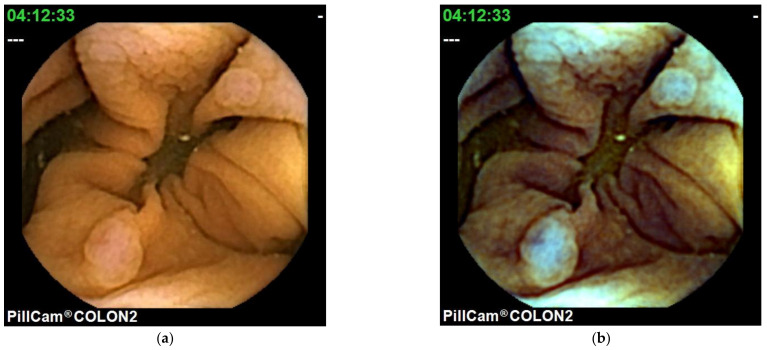

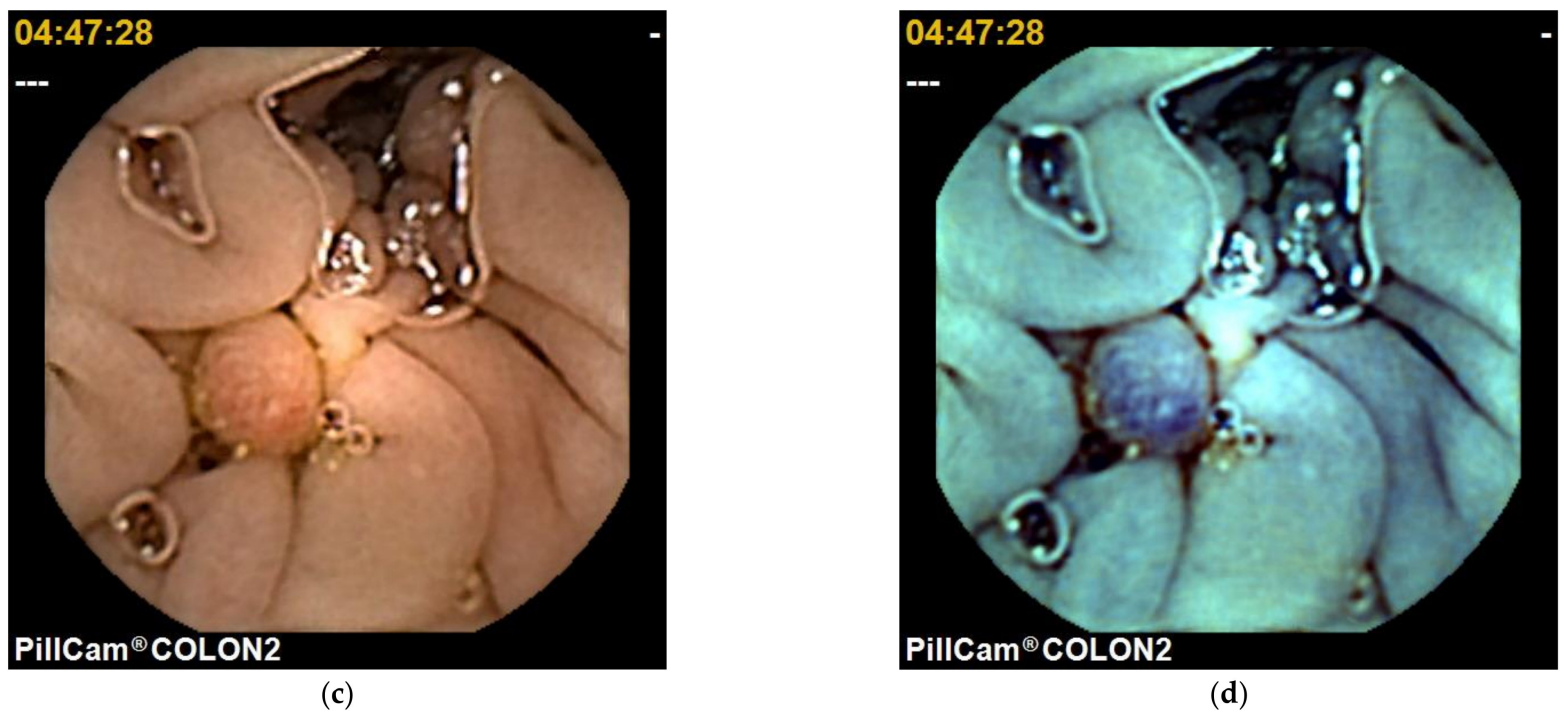
Capsule endoscopy images of colon capsule: WLI (**a**,**c**), FICE 1 (**b**,**d**).

Colon CE (CCE) was introduced in 2006 [44]. A colonoscopy is a standard tool for colorectal examination. CCE is presented as a noninvasive alternative method for screening colon cancer [45]. In 2009, second generation CCE (CCE 2), which has improved sensitivity, was developed [46]. CCE 2 has good accuracy in the detection of polyps and colorectal cancer [47] and is useful for patients with Crohn’s disease and ulcerative colitis [48,49,50,51,52,53]. CCE 2 is equipped with a FICE system. In CCE 2, only the FICE 1 mode can be used (Figure 3). Nakazawa et al. [54] showed that FICE 1 improved the ability to differentiate between adenomatous polyps and hyperplastic polyps compared to WLIs using the CIElab color space method. They investigated the ability of the ΔE to differentiate between adenomatous polyps and hyperplastic polyps. They reported that among 34 adenomatous polyps, the FICEΔE (39.2 ± 19.3) was significantly higher than the WLIΔE (14.9 ± 10.4) (*p* < 0.001). In contrast, among 17 hyperplastic polyps, the FICEΔE (12.8 ± 10.4) was not significantly different from the WLIΔE (10.7 ± 6.8) (*p* = 0.44). The FICEΔE of adenomatous polyps was 3.3 ± 1.8, which was significantly higher than the FICEΔE of hyperplastic polyps (1.3 ± 0.6; *p* < 0.001). Further large studies need to be conducted to confirm that FICE in CCE 2 is a highly useful modality for screening colon polyps.

## 7. Conclusions

In conclusion, FICE and CC appear to improve the detection rate and visibility of small intestinal lesions. Previous reports used mostly retrospective analysis; thus, in the future, large prospective trials are needed to clarify the efficacy of IECE.

## 8. Discussion

Although many reports showed the usefulness of IECE for small intestinal lesions, the benefits of using IECE for small intestinal lesions remain controversial. FICE 1 may be useful for angioectasias and ulcers/erosions in the delineation and the detection of lesions. Because FICE1 was determined to reduce the interference of bile, it appears to improve the visibility of small intestinal lesions. There are also conflicting reports for the utility of IECE. Furthermore, on meta-analysis, 13 studies (nine evaluated detection only; one evaluated detection and characterization; and three evaluated characterization only) were analyzed [55], and, overall, the use of the FICE did not significantly improve the visualization or the detection rate of small intestinal lesions on CE when compared to WLI, although FICE 1 performed better in improving the delineation and the detection of small intestinal lesions. For angioectasias viewed under FICE 2 and 3, and for mucosal ulcers/erosions viewed under all three FICE modes, less than 50% of the images were improved. Sometimes, the use of IECE may reduce the amount of light and make observation difficult. The image pixelation caused by CE remains remarkably low, compared with that of conventional high-definition gastrointestinal endoscopy. If a next generation CE with high definition is introduced, IECE will be more useful in detecting and diagnosing small intestinal lesions. Although this review focused on FICE and CC, other IECE techniques are currently available, including blue mode (BM, Fujifilm Corp., Tokyo, Japan) and Augmented Live-body Image Color-Spectrum Enhancement (ALICE) (Intromedic, Seoul, South Korea). Blue mode is an additional image enhancing technique available for use with the RAPID software. The blue mode wavelength setting is 490–430 nm. Krystallis et al. reported that blue mode offered better image enhancement in CE as compared with FICE [41]. However, Koulaouzidis et al. [56] reported that although blue mode may enhance mucosal details, i.e., small mucosal breaks, it did not perform better than WLI in the calculation of the Lewis score in their cohort. In their study, computational analysis of our CE database was performed to identify patients who underwent CE with PillCam and had fecal calprotectin (FC) measured within 30 days after CE with PillCam. Only patients who had undergone prior colonoscopy were included to exclude any colon pathology-associated FC increase. Each small bowel tertile was reviewed (viewing speed 8 fps) with WLI and blue mode, in a back-to-back mode, by a single experienced reviewer. The Lewis scores were calculated after evaluation of each WLI and blue mode. The Pearson rank correlation (rho, *r*) statistic was applied. Twenty-seven patients were included, of which 13 underwent CE with PillCam SB1 and the remainder (*n* = 14) with PillCam SB2. The median level of FC in this cohort was 125 μg/g. In the WLI CE review, the Lewis score correlation with FC levels was *r* = 0.490 (*p* = 0.01), while in blue mode, it was *r* = 0.472 (*p* = 0.013). Regarding the MiroCam software (IntroMedic, Soul, Korea), Ryu et al. showed that ALICE improved the visibility of flat and depressed small bowel lesions [57]. Ribeiro et al. [58] reported that another IEE system that enhances images in 3 color modes (CM 1, CM 2, and CM 3) was not useful to improve the evaluation and characterization of any of the small intestinal lesions. In their study, 22 patients were selected, in whom 100 elementary lesions were identified, including erosions (*n* = 45), ulcers (*n* = 17), and angioectasias (*n* = 38). For each lesion identified, images were captured without chromo endoscopy (WLI) and with CM 1, CM 2, and CM 3. A score of 1 to 4 was assigned to each image, classifying the characteristics and limits of the lesion in ascending order, where 1 was the worst and 4 the best. The scores obtained using various modes were compared with Kendall’s tau-c coefficient. The average scores attributed to the photographs from WLI, CM 1, CM 2, and CM 3 were 3.83, 2.89, 1.85, and 1.43, respectively (tau-c = −0.75, *p* < 0.001). Evaluating the elementary lesions independently, the average scores for WLI, CM 1, CM 2, and CM 3 were 3.83, 2.92, 1.86, and 1.38 (tau-c = −0.77, *p* < 0.001) for erosions, respectively; 3.87, 2.96, 1.76, and 1.40 (tau-c = −0.80, *p* < 0.001) for ulcers, respectively; and 3.81, 2.82, 1.87, and 1.50 (tau-c = −0.71, *p* < 0.001) for angioectasias, respectively. The European Society of Gastrointestinal Endoscopy does not recommend the routine use of virtual chromoendoscopy during evaluation of the capsule recording since it does not appear to improve lesion detection or characterization [59]. In the field of gastrointestinal endoscopy, artificial intelligence has been explored enthusiastically [60,61,62,63,64,65,66]. The application of computer-aided detection for IEE has been the area most eagerly investigated. In the field of CE, several studies reported artificial intelligence algorithms for identifying small intestinal lesions [67,68,69,70,71,72,73,74,75,76,77,78]. In addition, some studies reported that deep learning has achieved excellent performance for the detection of small intestinal lesions in CE. With larger study samples and prospective multicenter trials, it is expected that computer-aided detection will be applied to CE in the future.

## Figures and Tables

**Table 1:** Clinical studies involving image-enhanced capsule endoscopy for the detectability of small intestinal lesions.

Reference	Country	Reviewers	Videos	Detectability								
				Angioectasia			Erosion/Ulcer			Tumor		
				FICE1	FICE2	FICE3	FICE1	FICE2	FICE3	FICE1	FICE2	FICE3
Imagawa et al. [30]	Japan	2	50	↑	↑	→	→	→	→	→	→	→
Gupta et al. [31]	Belgium	2	60	→			→					
Duque et al. [34]	Portugal	4	20		↑			→			→	
Kobayashi et al. [32]	Japan	3	24	↑	→	→	↑	→	→	↓	→	→
Matsumura et al. [35]	Japan	2	81	→			↑			→		
Sakai et al. [36]	Japan	4	12	↑	↑	→	↑	↑	↑			
Konishi et al. [37]	Japan	5	10	↑	↑	→	↑	↑	→			
Nakamura et al. [38]	Japan	2	50		↑							
Sato et al. [33]	Japan	3	50	↑	↑	→	→	↑	→	→	→	→
				**CC**			**CC**			**CC**		
Ogata et al. [25]	Japan	2	24	↑			↑					
Hatogai et al. [39]	Japan	10	5							→		

Abbreviations: ↑; significant improved, →; no significant change, ↓; significant decreased.

**Table 2:** Clinical studies involving image-enhanced capsule endoscopy for the visibility of small intestinal lesions.

Reference	Country	Reviewers	Images	Visibility								
				Angioectasia			Erosion/Ulcer			Tumor		
				FICE1	FICE2	FICE3	FICE1	FICE2	FICE3	FICE1	FICE2	FICE3
Imagawa et al. [40]	Japan	5	145	↑	↑	→	↑	↑	→	↑	↑	→
Krystallis et al. [41]	UK	2	167	↑	→	→	↑	↓	↓	→	↓	↓
Sato et al. [33]	Japan	5	261	↑	↑	→	↑	↑	→	→	→	→
Cotter et al. [42]	Portugal	2	100	↑	↑	→	↑	↑	→			
				**CC**			**CC**			**CC**		
Ogata et al. [25]	Japan	3	138	↑			↑					
Hatogai et al. [39]	Japan	10	20							↑		

Abbreviations: ↑; significant improved, →; no significant change, ↓; significant decreased.

## Data Availability

Not applicable.
